# The Relationship between HIV Duration, Insulin Resistance and Diabetes Risk

**DOI:** 10.3390/ijerph18083926

**Published:** 2021-04-08

**Authors:** Eduard Tiozzo, Allan Rodriguez, Janet Konefal, Gary J. Farkas, Jennifer L. Maher, John E. Lewis

**Affiliations:** 1 Department of Physical Medicine and Rehabilitation, Miller School of Medicine, University of Miami, Miami, FL 33136, USA; gjf50@med.miami.edu; 2 Department of Medicine, Miller School of Medicine, University of Miami, Miami, FL 33136, USA; ARodriguez2@med.miami.edu; 3 Department of Family Medicine, Miller School of Medicine, University of Miami, Miami, FL 33136, USA; jkonefal@med.miami.edu; 4 Department for Health, University of Bath, Bath BA2 7AY, UK; jlm92@bath.ac.uk; 5 Department of Psychiatry & Behavioral Sciences, Miller School of Medicine, University of Miami, Miami, FL 33136, USA; JLewis2@med.miami.edu

**Keywords:** HIV infection, duration, HOMA-IR, insulin resistance, type 2 diabetes

## Abstract

The risk of developing Type 2 Diabetes Mellitus in people living with HIV (PLWH) can be four times greater and can occur at an earlier age and even without the presence of obesity compared to those without HIV. Therefore, the purpose of this analytical cross-sectional study was to determine the relationship between HIV duration and glucose metabolism among PLWH. Eighty-two PLWH were categorized into shorter (≤15 years) or longer HIV duration (≥16 years) and then compared for differences in demographics, physical and clinical characteristics, biomarkers, and dietary intake. Compared to those with shorter HIV duration (*n* = 34), those with longer HIV duration (*n* = 48) were on average older (*p* = 0.02), reported lower consumption of alcohol (*p* = 0.05), had higher levels of homeostasis model assessment of insulin resistance (HOMA-IR, *p* = 0.02), were also more likely to be a woman (*p* = 0.06), and have higher levels of fasting insulin (*p* = 0.06). When adjusted for age and body weight, the levels of HOMA-IR and fasting insulin were higher (*p* = 0.02 and *p* = 0.04) with longer compared to shorter HIV duration, respectively. Longer exposure to HIV infection is associated with impaired insulin sensitivity. Continuing research aimed at the long-term effects of HIV infection and (antiretroviral therapy) is required.

## 1. Introduction

With the use and advancement of antiretroviral therapy (ART), human immunodeficiency virus (HIV) has transitioned from a deadly disease to a chronic and manageable condition [1,2]. Marked improvements in ARTs potency and side-effect profile, coupled with their greater availability and ease of use, have led to a dramatic increase in life expectancy of people living with HIV (PLWH). In fact, life expectancy of PLWH is currently similar to that of the general population [3]. 

With an extended lifespan, PLWH and their health care professionals now face increased challenges for complex care of emerging non-communicable diseases, including Type 2 Diabetes Mellitus (T2DM). Our previous work [4] and other studies [5,6] have reported increased incidence and prevalence rates of metabolic syndrome and T2DM among PLWH. The risk of developing T2DM in PLWH, compared to those without HIV, can be up to four times greater [7] and can occur at an earlier age and without obesity [8]. The etiology of T2DM is multifactorial and besides conventional risk factors, the aging PLWH additionally deals with prolonged exposure to HIV and ART, as potential determinants of excess glycemic burden and overall T2DM risk [2,9].

The objective of this study was to determine the relationship between length of HIV diagnosis and glucose metabolism. Additionally, we compared diabetes rates among our cohort with the U.S adult diabetes rates.

## 2. Materials and Methods

Ninety male and female PLWH were enrolled in “Healthy Living for Better Days”, a one-year, healthy lifestyle community program targeting PLWH of low socioeconomic status and on stable ART. Participants were recruited from the Adult HIV Clinic at University of Miami (UM) Miller School of Medicine/Jackson Health Care System and other Miami-Dade County clinics. Eligible participants were those who: (1) had been diagnosed with HIV, (2) were ≥18 years of age, and (3) could attend weekly, in-clinic exercise sessions at UHealth Fitness and Wellness Center at the UM Miler School of Medicine. We excluded participants with any medical condition or disease for which exercise is contraindicated and pregnancy in women. Details of methods and assessments used in the program are described elsewhere [4,10]. The program was approved by the Institutional Review Board for human subject research at the UM. All participants gave written informed consent to participate in the program.

In addition to basic sociodemographic information, the following outcomes were included in the present study: (1) physical characteristics, including body weight, body mass index (BMI), waist circumference (WC), systolic blood pressure (SBP), and diastolic blood pressure (DBP); (2) glucose metabolism markers (fasting glucose [FG], fasting insulin, homeostasis model assessment of insulin resistance [HOMA-IR], and hemoglobin A1c [HbA1c]), were diabetes classification was based on the American Diabetes Association criteria (FG of ≥ 126 mg/dl and HbA1C ≥ 6.5%) and the study values were compared to the rates from the 2017 National Diabetes Statistics Report [11]; (3) lipid profile (total cholesterol [T-Chol], high-density lipoprotein cholesterol [HDL-C], low-density lipoprotein cholesterol [LDL-C], very-low density lipoprotein cholesterol [VLDL-C], and triglycerides [TGs]); (4) high-sensitivity C-reactive protein (hsCRP); and (5) a comprehensive nutritional assessment, using the Block Food Frequency Questionnaire (FFQ) [12]. 

Anthropometric measurements were assessed using standard techniques [4]. Weight and height were recorded to the nearest 0.1 kg and 0.1 cm, respectively, to calculate BMI. Waist was measured in inches at the narrowest circumference halfway between the lowest rib and the iliac crest. SBP and DBP were measured by use of an automatic oscillometric devise, Omron Blood Pressure monitor (Omron Healthcare, Inc., Kyoto, Japan). Three readings were made with the participants seated after resting their back against the chair for at last five minutes. The average of the second and third readings was used [13]. Data were also collected for lipid-lowering, antihypertensive, diabetes drugs, and current ART (protease inhibitors vs. non-protease inhibitors). Additional lifestyle data included self-reported smoking (none or less than a half pack, a half to one pack, and more than one pack per day), coffee consumption (none or average cups per day), alcohol consumption (none or average drinks per week), and sleep (average hours per night).

Blood was drawn during the morning in at least an 8-h fasted state. Chemistry and immunoassays were performed by an automated analyzer (Roche Cobas-6000; Roche Diagnostics, Indianapolis, Indiana). Serum was used to quantify hs-CRP in a high sensitivity latex-particle enhanced immunoturbidimetric assay with a detection limit of 0.1 mg/L with intra-assay and inter-assay coefficients of variations of 1.1% and 2.2%, respectively. T-Chol and TGs were determined in serum or plasma by enzymatic, colorimetric assays. Intra-assay and inter-assay coefficients of variation were 0.7% and 1.8%, respectively, for T-Chol and 0.9% and 2.3%, respectively, for TG. HDL-C was measured using a third-generation homogeneous enzymatic colorimetric assay, and the intra-assay and inter-assay coefficients of variations were 0.6% and 1.9%, respectively. LDL-C was calculated using the Friedewald formula: LDL-C = T-Chol − (HDL-C − 0.20 × TG). VLDL-C was calculated as TG divided by five, unless TG exceeded 400 mg/dL in which case it was measured by enzymatic methods (Vitros 750 Analyzer, Johnson & Johnson, New York, NY, USA). FG was measured by the hexokinase method with intra-assay and inter-assay coefficients of variations as 1.9% and 2.7%, respectively. Insulin was measured in fasting plasma or serum by electrochemiluminescence immunoassay on a Roche Cobas 6000 auto-analyzer using manufacturer’s reagents and protocols for instrument set up, operation, and analysis (Roche Diagnostics, Indianapolis, IN, USA). Assy measuring range is from 0.2 to 1000 µU/mL (1.39–6945 pmol/L) and the intra and inter assay CV are 1.4% and 3.2%, respectively. HOMA-IR was calculated according to the formula: fasting insulin (µU/L) × FG (nmol/L)/22.5 (35). HbA1c in whole blood was measured by high-performance liquid chromatography using a fully automated analyzer (Variant II Hemoglobin Testing System, BioRad, Richmond, CA, USA), and intra-assay and inter-assay coefficients of variations were 1.7% and 2.0%, respectively.

The Block FFQ was used to measure long-term dietary intake. Based on frequency and quantity of food intake “over the past year or so,” we estimated daily intake of: total calories (kcal), protein (g), total fat (g)-including saturated and trans fats-carbohydrate (g)-including total and added sugar/syrup to food and beverages-fiber (g), fruit and vegetable intake (cup), average daily glycemic load (g), and average daily glycemic index.

Descriptive statistics was calculated on all variables for participants with available HIV duration data (*n* = 82). To conduct the subsequent statistical analyses and to evaluate the objective of the study, participants were categorized into two groups based on duration of HIV infection: shorter duration (≤15 years) or longer duration (≥16 years). The two groups were then compared for differences in demographics, physical and clinical characteristics, biomarkers, and dietary intake with independent samples t-tests for continues variables and chi-square test of independence for categorical variables. Any significantly different variables were then compared with analysis of covariance (ANCOVA) where age and body weight were covariates in model 1 and BMI and WC were added as additional covariates in model 2. As HOMA-IR and fasting insulin level were significantly different between groups, we reported the percent changes in five-year increments of HIV exposure time for each variable. Additional analyses were conducted to determine if excluding the participants on the antidiabetic medications (*n* = 8) altered results. All statistical analyses yielded consistent results. Presented results include participants on the antidiabetic medications.

SPSS 26 (IBM Corporation, Armonk, New York, NY, USA) was used for all statistical analysis, and an α ≤ 0.05 was considered significant.

## 3. Results

The mean age of our participants (*n* = 82) was 48 ± 7 years; 54% were women, 67% African American, 23% Hispanic, and 10% non-Hispanic Caucasian. Most of our participants (80%) were not employed at the time of baseline assessment. The average number of years since HIV diagnoses was 16 ± 7, with 59% of individuals being exposed to HIV for ≥ 16 years (defined as longer HIV duration) and 41% of individuals for < 16 years (defined as shorter HIV duration). Compared to those with shorter HIV duration, those with longer HIV duration (Table 1) were on average older (+4 years; *p* = 0.02), reported lower consumption of alcohol (*p* = 0.05) and were more likely to be a woman (*p* = 0.06).

Table 2 presents biomarkers and dietary intake. The longer HIV duration group had borderline higher levels of insulin levels (*p* = 0.06) and significantly higher HOMA-IR (*p* = 0.02) than the shorter HIV duration group. Although not statistically significant, the longer HIV duration group showed higher levels of FG (*p* = 0.15) and HbA1C (*p* = 0.21). Lipids and hsCRP were similar between the groups. Although not statistically significant, those with longer HIV duration had lower self-reported daily caloric intake (−306 Kcal; *p* = 0.24), with the majority of the deficit coming from lower intake of carbohydrates (−148 Kcal, *p* = 0.27).

ANCOVAs were performed with both HOMA-IR and insulin as the dependent variables. In model 1, age and body weight were controlled, and in model 2 BMI and WC were additionally controlled to determine if duration of HIV exposure was still significant. In model 1, those with longer HIV duration still had significantly higher levels of HOMA-IR, than those with shorter HIV duration (mean 4.0 ± 2.8 vs. 2.8 ± 1.6; F [1, 78] = 6.00, *p* = 0.02). A similar finding was found for fasting insulin, where those with longer HIV duration had a significantly higher level compared to those with shorter HIV duration (mean 15.9 ± 10 vs. 12.4 ± 6.6; F [1, 78] = 4.40, *p* = 0.04). In model 2, HIV duration had no significant association with HOMA-IR (*p* = 0.08) and fasting insulin (*p* = 0.16) after full adjustment.

Overall, PLWH in our study had a higher rate of T2DM compared to the adult general population in the United States (15.9% vs. 12.2%, respectively) from the 2017 National Diabetes Statistics Report [11]. When our participants were divided into two groups, according to duration of HIV, the group with shorter exposure had a lower rate (8.8%), while the group with longer HIV exposure had a higher diabetes rate (20.8%). 

Table 3 presents the changes in HOMA-IR and fasting insulin in five-year increments, with the rise in both markers occurring between 10 and 15 years and remaining elevated afterward.

## 4. Discussion

In the present study, of PLWH of predominantly low socioeconomic status, longer exposure to HIV infection is associated with impaired insulin sensitivity as measured by HOMA-IR and fasting insulin. This finding is independent of some common risk factors, such as age and body weight but not BMI and WC. It has been inconclusive whether HIV infection and ART contribute to higher risks of insulin resistance and T2DM, independent of traditional risk factors such as age and body weight [14]. In our study, we demonstrated that specifically the duration of HIV infection, and consequently the use of ART, may further elucidate a possible metabolic dysregulation in PLWH.

The present study revealed a significant impact of HIV duration on HOMA-IR, as surrogate markers for insulin resistance and development of T2DM. The HOMA-IR model has been a widely used clinical and epidemiological tool in estimating insulin sensitivity and β-cell function from fasting insulin and glucose levels [15]. However, HOMA-IR has no agreed-upon cutoffs [16,17]. Different studies implement different cutoffs, making it difficult to ascertain prevalence and incidence rates of insulin resistance or to compare the rates across different populations. When the HOMA-IR cutoff of ≥2.35 is used, the prevalence estimate of insulin resistance in normoglycemic adults in the general US population is 32% [18]. Using the same cutoff, the prevalence of insulin resistance in our sample is 60%, with higher rates in the group with longer compared to shorter HIV duration (63% and 56%, respectively). A study in 265 PLWH [19] used a higher cutoff for insulin resistance (HOMA-IR ≥ 3.8) and estimated a 21% prevalence rate. Compared to the same HIV cohort and using the same HOMA-IR cutoff, our HIV cohort would have a 33% rate of insulin resistance. Again, we observed the differences based on the HIV duration, with longer HIV duration exhibiting markedly higher rate of insulin resistance, than shorter HIV duration (39% and 25%, respectively).

Fasting insulin and HbA1C represent a proxy marker of insulin resistance and diabetes risk. Unlike HbA1C, fasting insulins individual use has been limited due to low specificity and lack of standardization and reference values [20]. The studies show that high (plasma) insulin values in HIV-negative individuals, even with normal glucose tolerance, can indicate insulin resistance and presage the development of T2DM [21]. In our study, longer exposure to HIV led to a 28% higher average fasting insulin level, compared to shorter exposure. The same group difference was independent of age. This is interesting given that diminished hepatic insulin clearance and glucose sensitivity, thus higher insulin levels have been associated with aging [22]. HbA1C, an indicator of long-term glycemic control, and FG represent a standard of care for diagnosing and monitoring T2DM [20]. We did not observe significant differences in HbA1C and FG with different HIV duration, although their levels were 5% and 11% higher, respectively, in the longer exposure group.

In our study, HIV duration exposure was not associated with blood pressure, lipid profile, or hsCRP. Both exposure groups had identical average blood pressure levels, with their overall SBPs being slightly elevated and prehypertensive. Dyslipidemia has emerged as an important problem in PLWH receiving ART [23,24]. However, the lipid profile of our study participants and regardless of their HIV duration was similar, with all average cholesterol and triglyceride levels falling within normal reference ranges. While hsCRP was not different between groups, the hsCRP value of our entire study sample was elevated, indicating the presence of systemic inflammation and subsequent risk of cardiovascular events.

Despite reduced caloric intake, including natural and added sugar, the longer HIV group had greater insulin resistance-as measured by HOMA-IR-than the shorter HIV duration group. This association was independent of age and body weight but not BMI and WC. The longer HIV duration group had non-significantly higher BMI and WC, despite consuming less calories (and exhibiting lower muscular strength-not reported) than the shorter duration HIV group. The link between increased BMI, WC, and lower muscular strength (observed in the longer duration HIV group but not reported) with impaired insulin sensitivity has been established [25,26] and it is plausible that prolonged HIV infection coupled with aging may further exacerbate that association. The reason why the same association in our study was explained by age and weight and not BMI and WC might be related to the lack of accuracy in body composition assessment. As proxy measures of obesity, BMI and WC do not discriminate between skeletal muscle and body fat, which have opposite influences on diabetes risk [27]. Therefore, fat-free mass and percent body fat, as more precise physiological estimates of body composition, may be needed to fully elucidate which component(s) of body weight contribute(s) to a higher diabetes risk with longer HIV duration.

As far as the dietary differences, increased insulin resistance favors weight gain and/or prevents weight loss. That could explain why those with higher insulin resistance and longer HIV duration in our study had a general trend toward greater central adiposity and body weight, despite reduced calorie intake, including natural and added sugar intake. 

Overall, our PLWH cohort had higher rate of T2DM compared to the adult general population in the United States. However, more than that, our participants with longer HIV duration, compared to lower HIV duration, had higher rate of T2DM, which increases their risk for metabolic complications. In our previous work we also reported a comparable rate of metabolic syndrome between our cohort and the adult general population (33% and 34%, respectively) [4]. Considering that our study sample is of predominantly low socioeconomic status, it is conceivable that higher impairment of glucose metabolism seen in our current work is specific to our unique study demographics. Low socioeconomic status has been associated with higher pre-diabetes and diabetes rates and worse outcomes and mortality rates [28,29,30].

Strengths of the current study included a cohort with extensive information on diabetes risk factors, including dietary habits. While some study protocols had a 6–8 h fast before blood sampling, which could overestimate biomarkers, we utilized fasting for minimum 8 h. If participants consumed any calories on the morning of blood draw, they were re-scheduled and asked to adhere to the fast. Limitations of our study was a cross-sectional design with limiting inferences about temporality and causality. In addition, we did not assess the hepatitis C virus, which has been associated with insulin resistance and abnormal glucose tolerance in PLWH [31]. We also did not obtain HIV RNA level, CD4 cell count, and the duration of ART use to further examine the association between HIV duration and other outcomes of interest. Additionally, the assessment of body composition was performed through BMI and WC and not more precise measurements, such as percent body fat and lean body mass as measured by dual x-ray absorptiometry.

## 5. Conclusions

Our work indicates that longer duration of HIV should be explored as one of the risk factors of insulin resistance and T2DM. If satisfactorily proven, it would become even more relevant to offer and implement healthy lifestyle changes and treatments at the earliest stages of HIV infection, as well as routinely screen PLWH for pre-diabetes and diabetes. Such approach could only ameliorate the burden of insulin resistance and related non-communicable comorbidities, lately often observed among PLWH. Future research should continue to explore the long-term effects of HIV infection, its pathophysiology and pharmacology, on age- and non-HIV-related comorbidities. 

## Figures and Tables

**Table 1:** Demographic, Physical and Lifestyle Characteristics of Study Participants.

Variable	Category	Shorter HIV Duration <16 Years (*n* = 34)	Longer HIV Duration ≥16 Years (*n* = 48)	*p* Value
Age (years)	-	46 (9)	50 (5)	0.02 *
Sex *n* (%)	Female	14 (41)	30 (63)	0.06
	Male	20 (59)	18 (38)	
Ethnicity *n* (%)	African American	20 (59)	35 (73)	0.39
	Hispanic	10 (29)	9 (19)	
	Non-Hispanic, white	4 (12)	3 (6)	
Employment status *n* (%)	Disabled/Unemployed	28 (82)	39 (83)	0.94
	Working (Full- or part-time)	6 (18)	8 (17)	
Medications *n* (%)	PI	17 (55)	29 (60)	0.35
	Cholesterol-lowering	3 (10)	6 (13)	0.58
	Antidiabetic	2 (6)	8 (17)	0.14
	Antihypertensive	9 (29)	13 (27)	0.95
Physical Characteristics	SBP (mm/Hg)	126 (12)	126 (12)	0.96
	DBP (mm/Hg)	81 (9)	81 (9)	0.85
	BMI	30 (8)	32 (8)	0.28
	WC (inches)	40.5 (7)	42.9 (8)	0.15
Lifestyle habits	Current coffee drinker (*n*, %)	24 (71)	37 (77)	0.50
	Current alcohol drinker (*n*, %)	12 (35)	8 (17)	0.05 *
	Current smoker (*n*, %)	10 (29)	18 (38)	0.45
	Sleep (hours/night)	6.3 (1.8)	6.9 (1.7)	0.12

Data are mean (*SD*) or *n* (%), * Independent sample *t*-test, *p* < 0.05. PIs Protease Inhibitors, SBP Systolic Blood Pressure, DBP Diastolic Blood Pressure, BMI Body Mass Index, WC Waist Circumference.

**Table 2:** Biomarkers and Dietary Intake of Study Participants.

	Shorter HIV Duration ˂16 Years (*n* = 34)	Longer HIV Duration ≥16 Years (*n* = 48)	*p* Value
**Biomarkers**			
T-Chol (mg/dl)	187 (39)	183 (36)	0.63
LDL-C (mg/dl)	110 (35)	109 (32)	0.86
HDL-C (mg/dl)	52 (13)	51 (17)	0.66
VLDL-C (mg/dl)	25 (16)	24 (12)	0.65
T-Chol/HDL-C	3.8 (1.2)	3.9 (1.3)	0.65
Triglycerides (mg/dl)	125 (81)	117 (60)	0.64
hsCRP (mg/L)	5.9 (8.1)	6.1 (7.6)	0.93
Glucose (mg/dl)	91 (12)	100 (38)	0.15
HbA1C (%)	5.8 (0.8)	6.1 (7.6)	0.21
Insulin (uiU/mL)	12.4 (7)	15.9 (10)	0.06
HOMA-IR	2.8 (1.6)	4.0 (2.8)	0.02 *
Diet			
Total calories (Kcal)	2061 (1434)	1755 (924)	0.28
Total Fat (g)	82 (60)	69 (37)	0.27
Saturated Fat (g)	27 (21)	23 (12)	0.27
Trans Fat (g)	7 (8)	5 (5)	0.18
Protein (g)	83 (56)	73 (39)	0.34
Total Carbohydrates (g)	249 (181)	212 (122)	0.30
Sugar (g)	118 (94)	104 (70)	0.45
Added Sugar (g)	86 (83)	75 (67)	0.53
Fruit and Vegetable (cups)	3 (2)	3 (2)	0.98
Fiber (g)	13 (9)	12 (7)	0.46
Glycemic Load (g)	122 (88)	103 (62)	0.26
Glycemic Index	52 (4)	52 (5)	0.28

Data are mean (SD), * Two-sample t-test, *p* < 0.05. T-Chol Total Cholesterol, LDL-C Low-density Lipoprotein Cholesterol, HDL-C High-density Lipoprotein Cholesterol, VLDL-C Very-low density Lipoprotein Cholesterol, hsCRP High-sensitive C-reactive protein, HbA1C Hemoglobin A1c, HOMA-IR Homeostatic Model Assessment of Insulin Resistance, mg/dl milligrams per deciliter, uiU/mL international units per millilter.

**Table 3:** Five-Year Trends in HOMA-IR and Fasting Insulin Levels among Study Participants.

Variable	Category	*n* (%)	Mean ± *SD*	Range	Mean % Change from ≤5 Years
HOMA-IR	≤5 years	9 (11)	2.5 ± 1.3	1.2–5.5	
	6–10 years	9 (11)	2.2 ± 1.2	0.3–4.0	−12%
	10–15 years	16 (20)	3.3 ± 1.8	0.7–7.1	+32%
	15–20 years	24 (29)	3.2 ± 2.2	0.9–9.2	+28%
	20–25 years	13 (16)	6.1 ± 3.2	1.5–10.9	+141%
	>26 years	11 (13)	3.3 ± 2.2	0.8–7.5	+32%
Fasting Insulin	≤5 years	9 (11)	11.9 ± 6.9	5.9–27.7	
	6–10 years	9 (11)	9.4 ± 4.6	1.7–15.6	−21%
	10–15 years	16 (20)	14.4 ± 7.0	3.8–31.0	+21%
	15–20 years	24 (29)	13.5 ± 8.4	4.7–44.3	+13%
	20–25 years	13 (16)	21.9 ± 11.7	6.4–49.9	+84%
	>26 years	11 (13)	14.3 ± 9.0	3.2–30.7	+20%

HOMA-IR Homeostatic Model Assessment of Insulin Resistance.

## Data Availability

The data presented in this study are available on request from the corresponding author.
